# Exploration of the Genetic Organization of Morphological Modularity on the Mouse Mandible Using a Set of Interspecific Recombinant Congenic Strains Between C57BL/6 and Mice of the *Mus spretus* Species

**DOI:** 10.1534/g3.112.003285

**Published:** 2012-10-01

**Authors:** Gaëtan Burgio, Michel Baylac, Evelyne Heyer, Xavier Montagutelli

**Affiliations:** * Australian School of Advanced Medicine, Macquarie University, North Ryde, New South Wales 2019, Australia; † Origine, structure et évolution de la biodiversité, UMR 7205 and Plate-forme de Morphométrie, MNHN, CNRS-MNHN, UMS 2700, CP 50, Muséum National d’Histoire Naturelle, 75005 Paris, France; ‡ Unité d’éco-anthropologie, équipe « génétique des populations humaines », CNRS MNHN, P7, UMR 7206, CP 139, 75231 Paris, France; § Unité postulante de Génétique fonctionnelle de la Souris, CNRS URA 2578, Institut Pasteur, 75724 Paris, France

**Keywords:** quantitative trait loci, procrustes superimposition, modularity, morphological integration, mouse genetics, geometric morphometrics

## Abstract

Morphological integration and modularity within semi-autonomous modules are essential mechanisms for the evolution of morphological traits. However, the genetic makeup responsible for the control of variational modularity is still relatively unknown. In our study, we tested the hypothesis that the genetic variation for mandible shape clustered into two morphogenetic components: the alveolar group and the ascending ramus. We used the mouse as a model system to investigate genetics determinants of mandible shape. To do this, we used a combination of geometric morphometric tools and a set of 18 interspecific recombinant congenic strains (IRCS) derived from the distantly related species, *Mus spretus* SEG/Pas and *Mus musculus* C57BL/6. Quantitative trait loci (QTL) analysis comparing mandible morphometry between the C57BL/6 and the IRCSs identified 42 putative SEG/Pas segments responsible for the genetic variation. The magnitude of the QTL effects was dependent on the proportion of SEG/Pas genome inherited. Using a multivariate correlation coefficient adapted for modularity assessment and a two-block partial least squares analysis to explore the morphological integration, we found that these QTL clustered into two well-integrated morphogenetic groups, corresponding to the ascending ramus and the alveolar region. Together, these results provide evidence that the mouse mandible is subjected to genetic coordination in a modular manner.

The evolution of organism form is a complex process resulting from interactions among developmental, genetic, and environmental factors. The evolvability of such complex morphological features requires sophisticated mechanisms acting in coordinate manner. It is now well accepted that these morphological structures may be an assemblage of different semi-autonomous units (Atchley and Hall 1991; Chernoff and Magwene 1999; Cheverud 1996; Goswami and Polly 2010; Klingenberg 2008, 2010; Olson and Miller 1958; Wagner and Altenberg 1996; Wagner *et al.* 2007). Morphological integration is often defined as a composite association of traits driven by their developmental origins or functional requirements (Olson and Miller 1958). Modules often refer to units or a group of phenotypic traits that are independent and act in a coordinated manner (Klingenberg 2008, 2010; Olson and Miller 1958). Modularity is closely linked to morphological integration and corresponds to the degree of connectedness between modules (Wagner and Altenberg 1996). The genetic mechanisms underpinning morphological integration and modularity are still relatively unknown. One crucial question is to determine whether the genetic variation for shape is concentrated within modules rather than the morphological feature as a whole (Cheverud 1996; Klingenberg *et al.* 2004; Wagner and Altenberg 1996; Wagner *et al.* 2007).

Previously, studies have used the mouse mandible to uncover the genetic architecture for shape variation (Atchley *et al.* 1985; Bailey 1985; Festing 1973; Klingenberg *et al.* 2001; Leamy *et al.* 2008). Because of its functional and morphological complexity, it is considered as a valuable model to study the genetic architecture of complex traits (Klingenberg *et al.* 2001, 2004). During development, the shape of the mandible arises through a subtle imbrication between the cranial neural crest cells and the paraxial mesoderm; this process is under tight genetic control from growth and transcription factors, such as the Bmp4 or Dlx genes (Atchley 1993; Atchley and Hall 1991; Chai and Maxson 2006).

Significant insights into the genetic variation of the mouse mandible have been made using a quantitative genetic approach (Boell *et al.* 2011; Cheverud *et al.* 1997, 2004; Dohmoto *et al.* 2002; Ehrich *et al.* 2003; *et al.* 2001, 2004; Leamy *et al.* 2008; Suto 2009). Notably, these studies have provided strong evidence that the quantitative variation for shape is driven by a large number of quantitative trait loci (QTL) (Boell *et al.* 2011; Leamy *et al.* 2008; Navarro and Klingenberg 2007). These QTL act in a pleiotropic manner (Cheverud *et al.* 1997; Ehrich *et al.* 2003) associated with differential dominance, additivity (Ehrich *et al.* 2003), or epistatic buffering (Boell *et al.* 2011). Some reports have provided substantial evidence for defining the mandible into two morphogenetic components: the alveolar region and the ascending ramus (Atchley and Hall 1991; Atchley *et al.* 1985; Cheverud *et al.* 1997, 2004; Ehrich *et al.* 2003; Klingenberg *et al.* 2004). Notably, using Hooper’s squared trace correlation method [R^T^ coefficient (Hooper 1959)] on a second-generation cross between the LG/J and SM/J mouse strains, Klingenberg and colleagues have demonstrated that the genetic variation is not specifically clustered to the ascending ramus and the alveolar region modules; rather, it is more a relative degree of association between these two modules (Klingenberg *et al.* 2004). We sought to reassess the hypothesis of the variational modularity of the mouse mandible using a combination of a novel morphometric approach and a powerful resource based on an interspecific cross to enhance genetic and phenotypic polymorphisms.

The aim of this study is to determine whether the genetic variation for mandible shape is modular and integrated. To achieve this, we used a set of established mouse lines generated from an interspecific cross of the distantly related species, *Mus spretus* (the Mediterranean short-tailed mouse) and a classical inbred strain of mice, C57BL/6, largely derived from *Mus musculus domesticus* genome (Burgio *et al.* 2007). On average, 1.3% of the genome in each line contained SEG/Pas segments. The genomes of each line contained on average 1.37% SEG DNA, in the form of a few distributed chromosomal segments with an average size of 11 Mb [for more description on these lines see Burgio *et al.* (2007)]. These lines provide significant power for quantitative genetics studies because of the large evolutionary distance between the two genomes (Burgio *et al.* 2007). These lines have previously been used to identify novel quantitative loci governing hematological traits (Burgio *et al.* 2007), fertility traits (L’Hote *et al.* 2007, 2008, 2010, 2011; Laissue *et al.* 2009), hematologic disorders (Santos *et al.* 2009, 2010), and skull shape variation (Burgio *et al.* 2009, 2012) [for more details, see the review Dejager *et al.* (2009)].

In this study, we used geometric morphometric methods to quantify the modular variation of the mouse mandible. We have also conducted quantitative genetic studies to identify QTL associated with mandible shape variation. First, using a Procrustres ANOVA and variance component approach, we identified a bipartition of the genetic variation onto the ascending ramus and the alveolar region. We also showed that the 18 interspecific recombinant congenic strains exhibit shape differences compared with C57BL/6 and that the magnitude of shape variation is dependent on the proportion of SEG genome inherited but not on gene density. Using a multivariate correlation coefficient adapted for modularity assessment called the RV coefficient (Klingenberg 2009) and exploration of the morphological integration using a two-block partial least squares analysis, we showed bipartition of the mandible in two integrated morphogenetic components that supports the current modularity hypothesis. Together, these results provide evidence that the mouse mandible is subjected to a genetic coordination in a modular and coordinate manner.

## Materials and Methods

### Mice

The interspecific recombinant congenic strains (IRCS) are a set of 55 inbred lines derived from the introgression of the *Mus spretus*-derived SEG/Pas (SEG) strain (donor strain) into C57BL/6 (B6) genome as a recipient strain and selected on fertility traits (Burgio *et al.* 2007; L’Hote *et al.* 2007). This collection carries between 0 and 3.8% (average 1.37%) of the SEG genome on a B6 background. The *M. spretus* genome segments are distributed as a few chromosomal segments with an average size of 11 Mb (Burgio *et al.* 2007). Description of these strains was previously published (Burgio *et al.* 2007), and maps of genotypes are available at http://www.pasteur.fr/recherche/unites/Gfons/ircs/ircshome.htm. All animals were raised and housed in the same animal facility room, subjected to a 12:12 light:dark cycle, and received the same food (A03/10 pellets, SAFE, Augy, France). All protocols were in agreement with the Pasteur Institute guidelines for experiments on live vertebrates. A total of 362 males were used in this study from 18 IRCS lines chosen randomly from the 55 lines and C57BL/6 as a control (Table 1). For these analyses, the phenotype of the SEG strain differed from the IRCS lines to such an extent (*i.e.* a constant outlier) that the parental line was excluded from the study (data not shown).

**Table 1:** Number of mice, size, number of SEG segments and number of genes by IRCS

Strains	Number of Mice	Size of SEG Genome (Mb)	Number of SEG Segments	Number of Genes
C57BL/6	27			
5A	24	70.7	2	820
6A	20	65	3	594
6C	26	92.28	5	713
49A	14	65.75	3	546
66H	17	51.31	3	312
103E	9	37.82	4	324
119H	22	42.3	2	456
120C	20	108.5	5	850
122C	22	46.12	2	429
122D	14	73.19	4	937
122F	9	21.79	2	257
135B	20	49.58	2	333
135E	23	17.41	1	186
137E	24	17.1	1	192
137F	17	46.01	2	366
137G	21	27.38	2	264
157F	24	40.61	2	455
157D	9	9.97	1	124
TOTAL	362			

The number of genes encompassing the physical interval was determined using the Mouse Genome Informatics website (http://www.informatics.jax.org/genes.shtml).

### Data acquisition

**Figure 1:**
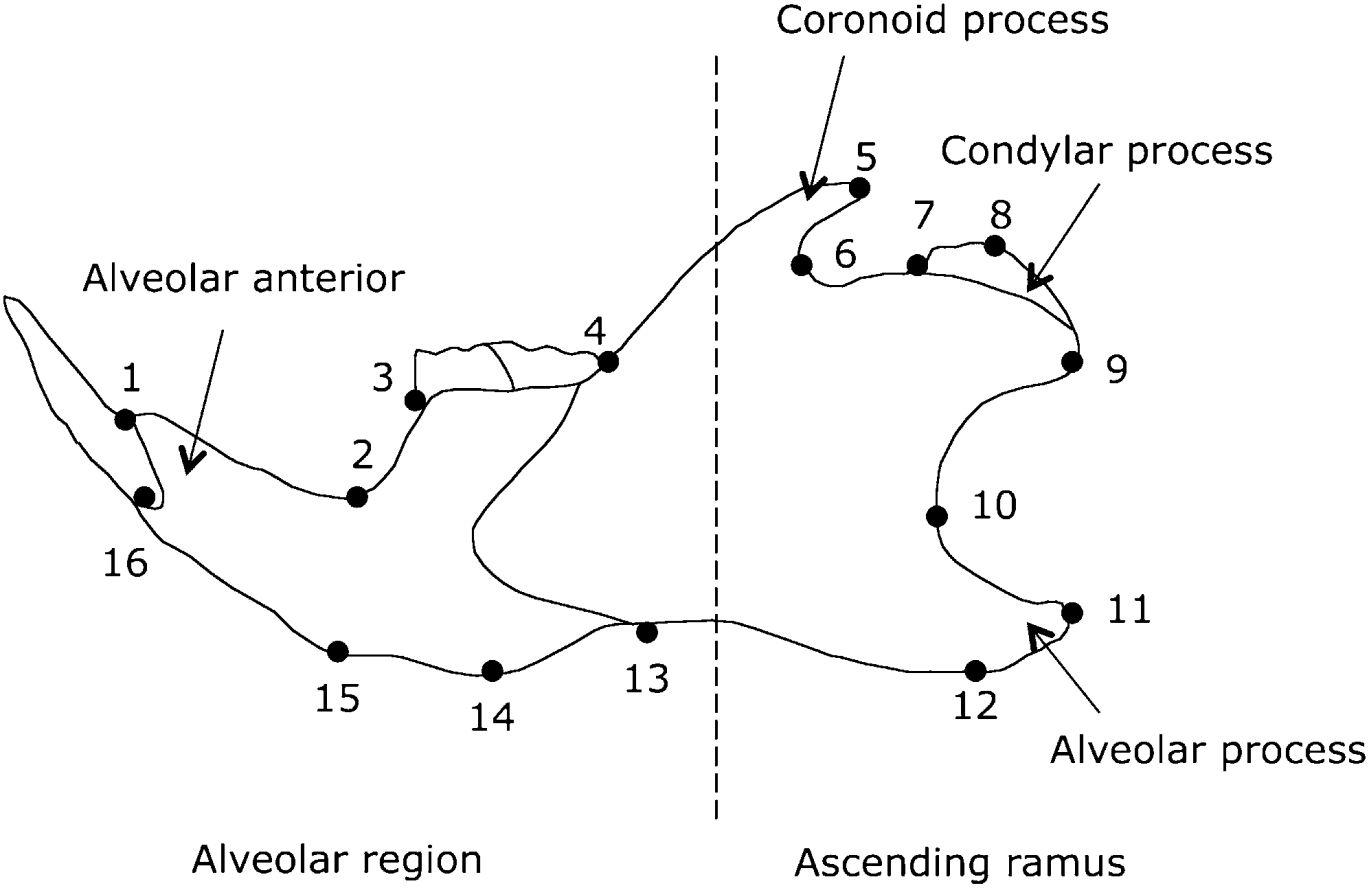
Mandible landmarks sampled on the IRCSs. Sixteen landmarks were digitized around the mandible outline. The dashed line divides the morphogenetic component into the alveolar anterior (landmarks 1–4 and 13–16) and the ascending ramus (landmarks 5–12).

All mice analyzed for mandible morphology were 60 ± 5-day-old males. The left and the right side of the mandible were separated at the mandibular symphysis. To study the genetic architecture of the mandible shape, we used landmark-based geometric shape analysis. The two-dimensional morphological landmarks were captured under a stereomicroscope (Nikon SMZ1500, Tochigi, Japan) using a 1.34 Mpixel digital camera (Axiocam HR, Carl Zeiss, OberKochen, Germany) and Axiovision 3.0 software (Carl Zeiss). Two acquisitions were obtained for each side of the mandible using Tpsdig 1.4 software (F.J Rohlf: http://life.bio.sunysb.edu/morph/soft-dataacq.html). For reproducibility, we defined 16 homologous landmarks around the mandible outline. Descriptions of the landmarks are shown in Figure 1.

### Morphometric analysis

To identify the QTL effects on shape changes and to interpret them in direct relation to the anatomy of the mandible, we carried out a geometric morphometric analysis. We performed a generalized Procrustes analysis (GPA) (Bookstein 1996; Dryden and Mardia 1998) that uses the generalized least-squares method. Two-dimensional coordinate points were translated, size standardized by the centroid size, and rotated. The mandibles were also superimposed using the matching symmetry procedure (Klingenberg and Mcintyre 1998) to pull out the asymmetric component for the genetic variation in these analyses. This procedure reflects all configurations from one side to their mirror images (Klingenberg and Mcintyre 1998). Symmetrized configurations were calculated as the mean of the left and right sides after matching and Procrustes superimposition. Asymmetric configurations were not included in the study. Measurement error and quantification of variation were assessed with a Procrustes ANOVA (Klingenberg and Mcintyre 1998). In these ANOVAs, the main effect of strain and individual gives an indication for variation of shape, whereas the main effect of side also indicates directional asymmetry. The side × individual interaction stands for fluctuating asymmetry. Then, the residual variance among variance components indicates a measurement error. To quantify the variation of these effects on the mandible shape, we carried out an analysis of the component of the variance for each landmark (Klingenberg and Mcintyre 1998) by the decomposition of the Procrustes mean squares for the strain, individuals, side, interaction between individuals and side, and residual effects in the ANOVA. Thus we summed *x* and *y* mean squares of each landmark separately and computed the variance components according to the expected mean squares (Klingenberg and Mcintyre 1998; Sokal and Rohlf 1995). The two-dimensional Procrustes superimposition procedure eliminates four degrees of freedom (reflection, translation, rotation, and size standardization). The relatively small strain sample sizes *vs.* the large number of variables could adversely affect the stability of the statistical analysis. To solve this issue, there are two alternatives. The first one is to reduce to 28 raw tangent coordinates [*e.g.* Leamy *et al.* (2008)]; the second is to perform a principal component analysis (PCA) on the covariance matrix of the tangent Procrustes coordinates to reduce the number of variables (Burgio *et al.* 2009; Friess and Baylac 2003; Monti *et al.* 2001). We opted for the second solution according to Monti *et al.* (2001) and Klingenberg *et al.* (2010). For all analyses (except the Procrustes ANOVA), we retained the 14 first principal components (PC), and in all cases, these axes accounted for over 90% of the total variance.

### Quantitative trait loci detection for mandible shape

After Procrustes superimposition, we sought to detect the genomic locations responsible for the genetic variation in this set of IRCSs. For this purpose, we carried out a multivariate analysis of variance (MANOVA) to assess the differences in shape between the IRCSs and B6, considering that the IRCSs carry close to 95% of the B6 genome. Eighteen MANOVA tests were conducted on the mandible shape coordinates (represented by the 14 PCs of the tangent coordinates) with strains as a covariate. *P* values were corrected with a Bonferroni adjustment, and the cutoff for a significant value was *P* < 0.002.

### Investigation of the relation between QTL effects and SEG segment length

We sought to assess whether the genetic variation of the mandible shape is correlated with the length of SEG segments or the number of genes. We conducted correlation tests between either SEG fragment lengths or the number of genes encompassed within the physical intervals for each strain (Table 1) and the QTL effects measured with the Procrustes distances between an IRCS and B6.

### Variation among IRCSs for the mandible shape

Next we sought to assess the among strains component of the genetic variation of mandible shape. We performed a canonical variate analysis (CVA) on the IRCSs and B6 to study the interstrains variation based from the 14 PCs on the tangent coordinates. A multivariate regression between the first two CVA axes and the corresponding Procrustes coordinates in a tangent space was performed to visualize shape changes on the CVA axes. Classification rates were calculated by the leave-one-out cross-validation procedure (Lachenbruch and Michey 1968). To visualize the genetic variation for mandible shape, the mean shape coordinates were calculated and superposed for the IRCSs and B6.

### Modularity assessment using RV coefficient

In this set of experiments, we aimed to determine whether the genetic variation of mandible shape is modular. To address this biological question, two *a priori* mandible modules were designated, corresponding to the alveolar region (landmarks 1, 2, 3, 4, 13, 14, 15, and 16) and the ascending ramus (landmarks 5–12) as previously reported (Atchley and Hall 1991; Klingenberg *et al.* 2004). To assess the level of modularity on the mandible, we measured the correlation between these two modules by applying the Escoufier RV coefficient (Escoufier 1973) on superimposed mandibles in the tangent space (Klingenberg 2009). The RV coefficient is a measure of the correlation between two or more sets of landmark coordinates and a multivariate generalization of the correlation coefficient R^2^. The RV coefficient is computed as the trace of the squared covariance matrix between the two sets of landmarks scaled by the square root of the squared variance and covariance within the two partitions (Klingenberg 2009). The hypothesis of the partition within the mandible was assessed by the comparison of RV coefficients for the subset to the total landmarks in the *a priori* and alternative contiguous partitions [see Klingenberg (2009) for a detailed discussion]. This corresponded to 6435 total partitions, including 94 contiguous partitions implemented in MorphoJ (Klingenberg 2011). A low observed RV value in the left tail of the distribution indicated a significant partition of the two subsets of landmarks.

### Two-block partial least-squares analysis

To investigate the degree of association between these two *a priori* modules, we carried out a two-block partial least-squares analysis (2B-PLS) (Rohlf and Corti 2000). Briefly, this analysis study the degree of covariance between two sets of landmarks by decomposing the covariance matrix of these *a priori* modules (*e.g.* alveolar region and ascending ramus) using a singular-value decomposition (Rohlf and Corti 2000). The original set of landmarks was then transformed into a pair of linear combinations of the two sets of variables. These pairs of linear combinations accounted for the largest amount of the covariance between these two sets of landmarks (Rohlf and Corti 2000). For this analysis, we only limited our analysis on the first pair of linear combinations of the two blocks, which in most cases, accounted for over 50% of the total covariance and correlation between the two sets of landmarks as previously described (Bookstein *et al.* 2003; Rohlf and Corti 2000). To assess the observed singular-value decomposition and correlations, 10,000 permutation tests were performed. A multivariate regression between the PLS blocks on the first axis and the corresponding Procrustes coordinates in a tangent space was performed to visualize shape changes into the first pair of PLS axis.

All the statistical analysis was carried out with MorphoJ (Klingenberg 2011) and the prereleased version of Rmorph library (Michel Baylac; baylac@mnhn.fr) under R v.2.7.0 (http://www.r-project.org). Additional programming was performed under R using MASS library (Gaëtan Burgio).

## Results

### Quantification of the amount of variation for mandible shape

To determine whether the mandible shape variation in this set of IRCSs was clustered to a specific part or to the mandible as a whole, we first quantified the strain, individual, and asymmetry effects on the amount of total shape variation of the mandible using Procrustes ANOVA. The results of the Procrustes ANOVA are summarized in Table 2. Directional asymmetry and fluctuating asymmetry were significant but contributed to only 5% and 4.1% of the total variance, respectively. Variation among strains and individuals accounted for 77.5% and 6.5% for the total mandible variation, respectively. Together, these results indicate that the strain effect was predominant, suggesting a dramatic genetic effect on shape variation. The analysis of the component of variance is an extension of the Procrustes ANOVA. It assesses the contribution of each landmark to mandible variation. The results are shown in the Table 3. Variance components of the side effect were evenly distributed among the landmarks. However, a large amount of the variation for the strain effect was distributed from landmarks 5 to 12, which are localized on the ascending ramus (81.5% of total variance). The variation on landmarks 5–12 contributed to most of the total variance for individuals, individual × side, or measurement error (76%, 72% and 82.6%, respectively). The coronoid process (landmark 5) and the angular process (landmarks 10, 11, and 12) were highly subjected to variation in shape. Together, these results indicate that a large amount of the mandible variation in this set of IRCSs is clearly distributed on the ascending ramus, suggesting the bipartition of the mandible variation into the ascending ramus and the alveolar region.

**Table 2:** Procrustes ANOVA on the mandible

	Sum of Squares	df	Mean Square	Variance Components	% Variance
Individuals (I)	0.775	9548	8.1 × 10^−5^***	19.66	6.49
Strain	0.476	504	0.0009***	234.75	77.52
Side (S)	0.194	28	0.0069***	20.34	6.71
I × S	0.422	10,052	4.2 × 10^−5^***	15.38	5.07
Error	0.256	19,992	1.26 × 10^−5^	12.66	4.18

Procrustes analysis of variance of the amount of shape variation attributable to different sources. Sum of squares, mean square, and variance components are in units of squared Procrustes distances (variance components × 10^6^). The percentage contribution (% variance) of each variance component to the total variance also is given. I stands for individuals, and side (S) indicates directional asymmetry. The interaction I × S stands for fluctuating asymmetry, and the residual variance indicates the measurement error. ^***^P < 0.001.

**Table 3:** Variance components for the effects in the Procrustes ANOVA listed by landmark

	Effect				
Landmark	Individuals	Strain	Side	Individuals × Side	Error
1	4	56	22	6	1
2	3	45	1	7	9
3	2	37	2	3	1
4	3	74	7	5	2
5	11	403	37	26	1
6	5	165	12	6	3
7	8	159	8	16	2
8	5	132	2	10	5
9	4	108	0	8	2
10	18	324	3	2	23
11	10	199	6	12	2
12	13	313	33	19	29
13	5	103	11	16	26
14	3	94	3	6	2
15	1	95	44	7	3
16	2	44	8	4	2

All entries have been multiplied by 10^7^ to make them more readable.

### Quantitative trait loci detection for mandible shape

Next, we sought to identify the genetic factors contributing to the variation of mandible shape. We carried out MANOVA between the IRCSs and B6, with strain as covariate (Table 4). All strains exhibited significant differences in mandible shape compared with the B6 reference strain. Moreover, some strains, such as 120C, 66H, or 157D, showed a high level of significance, suggesting a major genetic-based contribution. Out of 46 unique SEG segments, 42 were introgressed into this set of IRCSs, suggesting at most a detection of 42 QTL with only 25% of the SEG genome coverage. To refine this QTL detection, we further studied the strains carrying only one SEG segment and the IRCSs that have the same SEG fragment in common. The mandible shape of the congenic strains (135E: chromosome 19, 45–60 Mb from the centromere; 137E: chromosome 6, 135–160 Mb from the centromere; and 157D: chromosome 6, 140–160 Mb from the centromere) exhibited significant differences from B6, suggesting 3 different QTL in these genomic intervals. Some of the stains have a SEG DNA segment in common. For instance, the telomeric region of chromosome 6 is shared by the strains 137E (supporting information, Figure S1A), 157D (Figure S1B), and 157F (Figure S1C). To test whether a unique quantitative trait locus is responsible for the variation in mandible shape for these lines, we performed MANOVA tests on the reduced Procrustes coordinates between the congenic strain 157D and the two other IRCSs; only the difference between 157F and 157D remained significant (Wilks = 0.23, F_(14,18)_ = 4.28, *P* = 0.002). This result indicated that in the 137E and 157D strains, mandible shape variation is explained by a unique and common quantitative trait locus localized on chromosome 6 (140–160 Mb from the centromere), whereas the difference in shape for 157F is explained by an additional loci on chromosome 16 (0–20 Mb from the centromere). Therefore 2 QTL localized on chromosome 6 (140–160 Mb) and chromosome 16 (0–20 Mb) explained the variation in mandible shape for the strains 157F, 157D, and 137E. We have also concentrated our analyses on strains 103E (Figure S2A) and 6A (Figure S2B) that have a SEG fragment in common localized on the chromosome 19 (15–25 Mb from the centromere). To determine whether a common QTL explain the mandible shape variation for these two IRCSs, we carried out a MANOVA on the 14 PCs after Procrustes superimposition and found a significant difference in shape between 6A and 103E (Wilks = 0.045, F_(14,28)_ = 20.99, *P* < 0.0001), suggesting that the additional SEG segments explain the phenotypic variation of 6A and 103E. After refinement of the QTL detection, we found that 42 QTL spread into 18 IRCSs could still explain the genetic variation of these mouse lines.

**Table 4:** Multivariate analysis of variance for shape changes in IRCSs

Strain	Mandible				
	df Num	df Den	Wilks	F	*P*
5A	14	49	0.292	6.23	<0.0001
6A	14	45	0.174	10.81	<0.0001
6C	14	51	0.208	10.3	<0.0001
49A	14	39	0.057	30.3	<0.0001
66H	14	42	0.052	37.74	<0.0001
103E	14	34	0.14	8.92	0.0006
119H	14	47	0.153	13.39	<0.0001
120C	14	45	0.02	89.49	<0.0001
122C	14	47	0.111	19.35	<0.0001
122D	14	39	0.23	6.19	<0.0001
122F	14	34	0.067	20.71	<0.0001
135B	14	45	0.159	12.05	<0.0001
135E	14	48	0.409	3.67	0.0008
137E	14	49	0.131	17.01	<0.0001
137F	14	42	0.232	6.83	<0.0001
137G	14	46	0.092	21.83	<0.0001
157F	14	49	0.242	8.05	<0.0001
157D	14	34	0.05	28.15	<0.0001

MANOVA tests for differences in shape by testing within group (*i.e.* an IRCS strain with C57BL/6). Differences in shape are calculated using a multivariate regression in which the independent variable is the strain, and the dependent variables are 14 first principal components of the tangent coordinates in the shape space. *P* values are corrected with a Bonferroni adjustment.

### Visualization of the shape differences between IRCSs and B6

**Figure 2:**
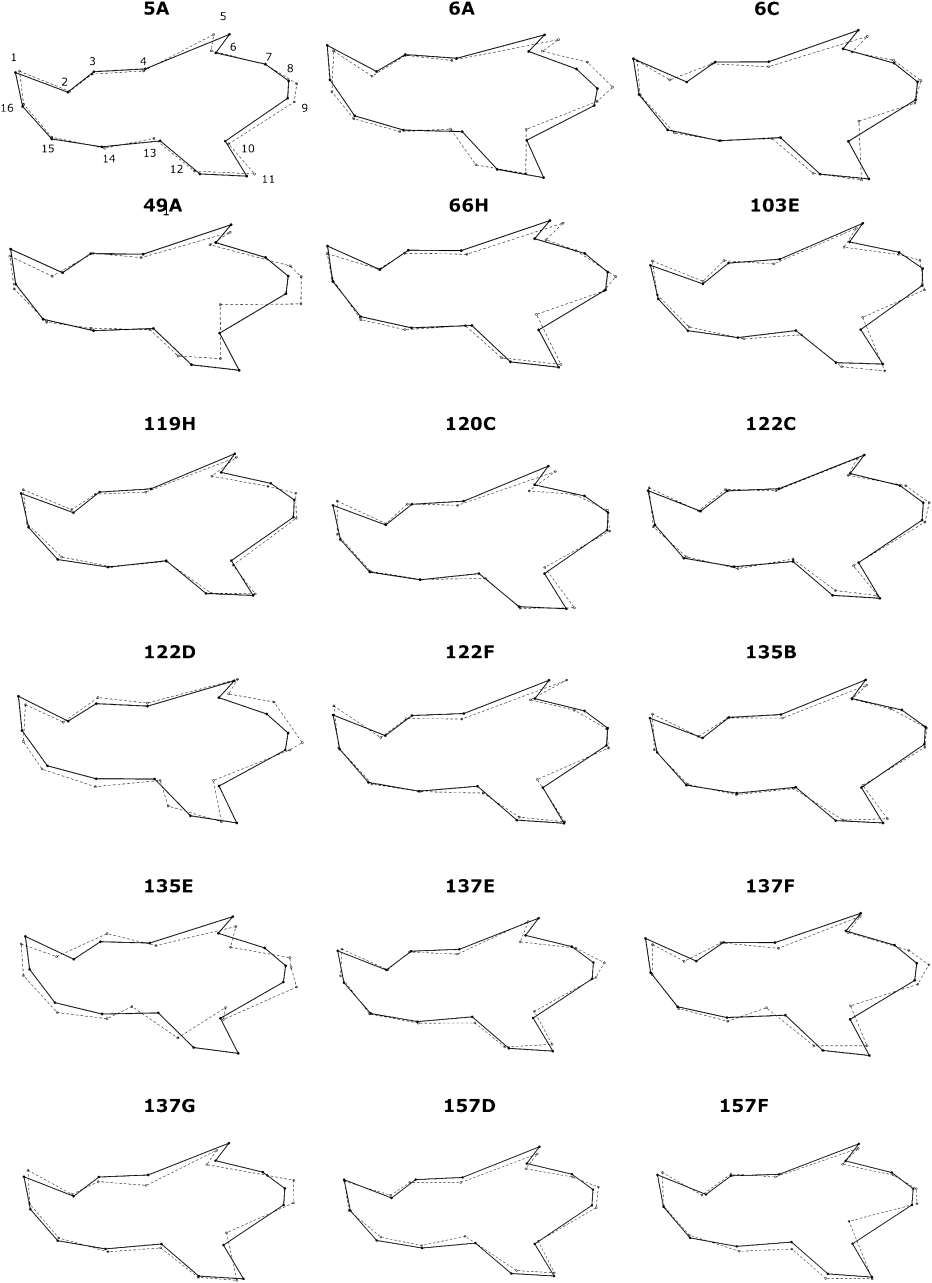
Visualization of the mandible shape of the IRCSs compared with C57BL/6. In each diagram, the landmarks are indicated by dots. The solid lines represent the mean shape for C57BL/6, and the dashed lines represent the mean shape for the IRCSs, indicating the spatial repartition of the effects of the introgressed SEG genome (as a form of IRCSs) on the mandible shape. Because the shape differences are subtle, all have been amplified by a factor of five.

We next sought to explore the spatial patterning of the significant IRCSs after MANOVA testing. Visualization of these shape differences between the IRCSs and B6 is shown in the Figure 2. Consistent with our previous analysis based on the Procrustres ANOVA, the main effects of the QTL were localized on the ascending ramus and moved the landmarks 8 to 10 on the antero-posterior axis. Additionally, we observed a marked shift on landmarks 5, 11, and 12 located on the angular and the coronoid process. On the molar alveolar or the incisor alveolar, a lengthening of the mandible in the supero-inferior axis characterized displacement of landmarks 2–4 and 13–15. Overall, the most striking observation was a shift and displacement of landmarks 5–12, along with only very modest effects on the alveolar region (landmarks 1–4 and 13–16) in some of the IRCSs (5A, 6A, 6C, 49A, 66H, 122D, 137G, and 157F). Interestingly, the shape changes for the congenic strains 135E, 137E, and 157D affected the mandible as a whole. Moreover, the shape changes for the IRCSs carrying the highest SEG genome rate (120C, 122D, 6C, 5A, and 49A) in our dataset were widespread throughout the mandible. Overall, the changes affected all parts of the mandible with a specific tropism on the ascending ramus.

### Exploration of the additivity of the genetic loci

**Figure 3:**
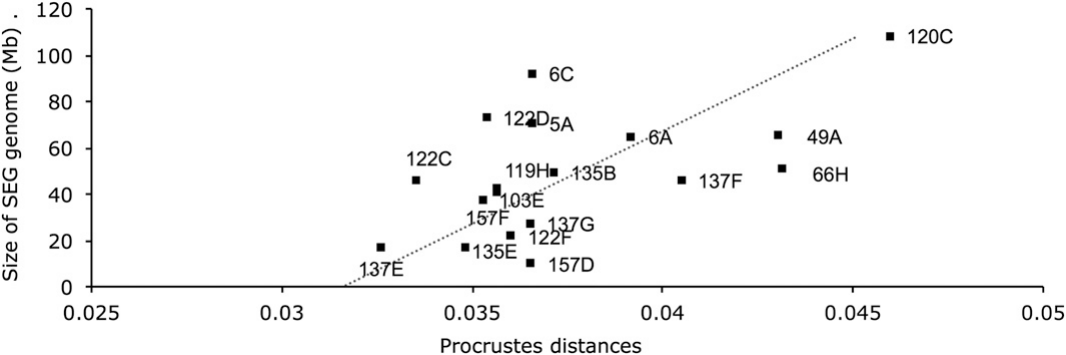
Correlation between the magnitude of Procrustes distances and the size of SEG segments on the mandible. The X-axis represents the Procrustres distances and the Y-axis represents the size of SEG genome per strain in megabases. The dashed line indicates significant correlation between the Procrustes distances and length of SEG segments.

We then hypothesized that the SEG segment length in each IRCS is responsible for the variation in shape compared with B6. To investigate this postulate, we plotted the QTL effects per strain measured with the Procrustes distances against the SEG segment length or the gene density for each IRCS (Figure 3). The IRCSs were distributed upon a line extending from 137E to 120C. Interestingly, the SEG segments localized in the strains 6C and 122D have little magnitude effect on shape variation. Conversely, the QTL localized in the strains 49A and 66H exhibited major effects in magnitude, suggesting a major gene effect. Overall, a strong correlation was observed between the Procrustes distances and the SEG segment length (r = 0.56, *P* = 0.01), but not with gene density (r = 0.3, *P* = 0.2).

### Among-strains variation for mandible shape variation

**Figure 4:**
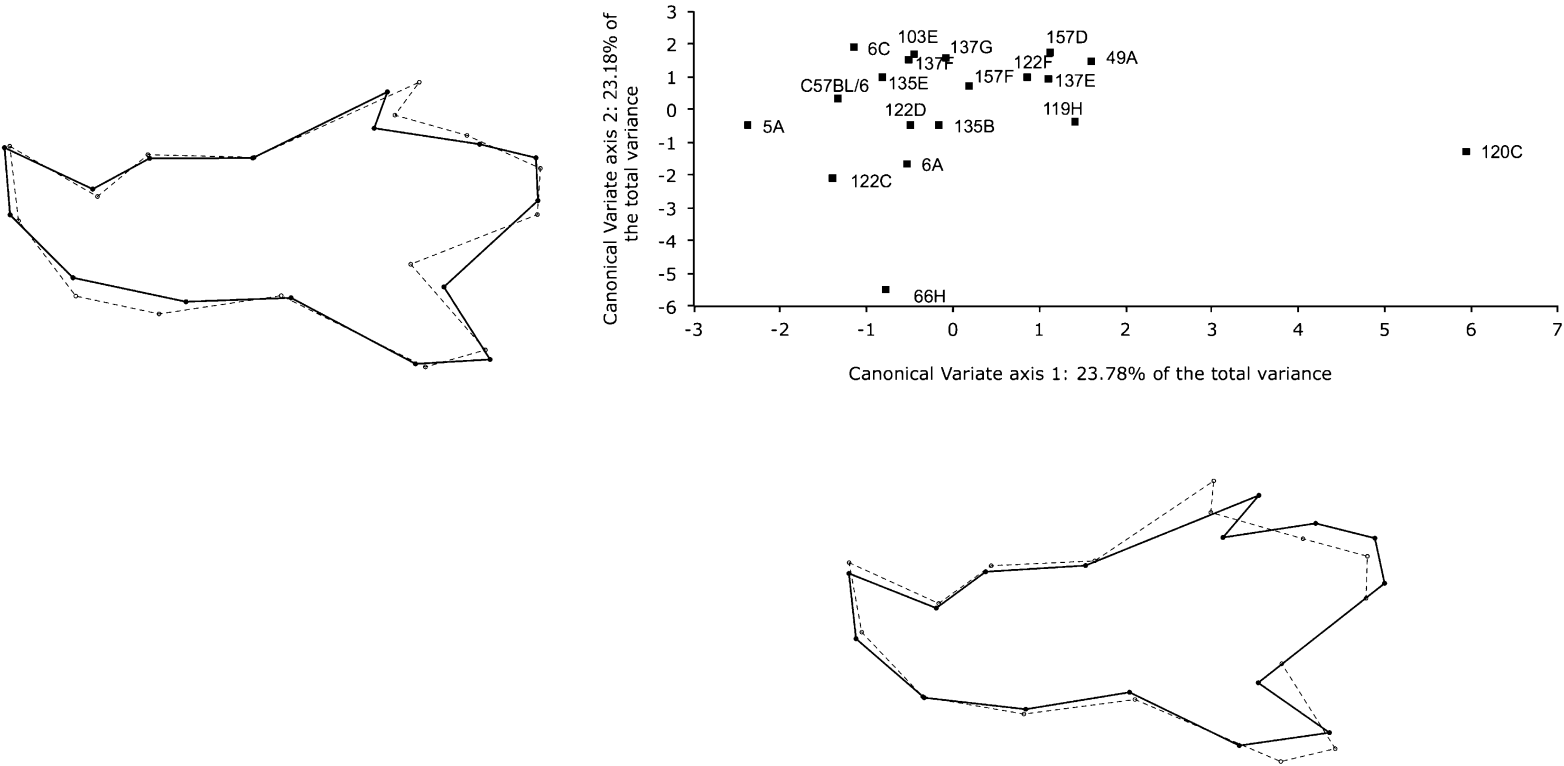
Comparison of mandible shape of the IRCSs by canonical variate analysis based on 14 first-principal components axes after Procrustes superimposition. The scatter plot corresponds to the canonical variate scores averaged by strain. The first and the second axes are represented, totaling 46.9% of the shape variation. Shape changes drawn outside of the scatter plot were calculated with a multivariate regression between the tangent coordinates after Procrustes superimposition and the first or second canonical axes. For each strain, the landmarks are indicated by dots; the solid lines represent the conformation corresponding to the higher value, whereas the dashed lines represent the shape changes for the lower value on the canonical axis. Because the effects were subtle, shape changes were magnified by a factor of two.

To study the differences among the IRCSs, we conducted a CVA based on 14 first PCs totaling over 90% of the total shape variation. Figure 4 depicts the result of the CVA analysis. The first and second canonical axes accounted for 46.96% of the total variation. Consistent with our previous analysis, line 120C exhibited a profound difference to other IRCSs and B6 on the first canonical axis compared with the other strains. The shape changes for the line 120C on the first canonical axis are characterized by an elongation of the ascending ramus, a shift of landmark 5 on the coronoid process, and a shortening of landmarks 11 and 12. Other lines (66H, 122C, 49A, and 119H) exhibited differences with B6 on the second canonical axis. These mandible shapes on the second canonical axes for these four IRCSs exhibited a displacement of landmarks 5–12 on the posterior ramus and landmarks 14 and 15. By contrast, the strains 135E, 103E, and 157F largely overlapped with B6 on both canonical axes. Leave-one-out cross-validation procedures were conducted to assess the correct classification for these IRCSs. The correct classification rate for the IRCSs was 70%. Only 60% of the B6 mandible mice were correctly classified. The B6 mandibles were misclassified with 135E (22%), 6A (7%), and 119H, 122C, and 137F (13%). Overall, the variation among strains mostly affected the ascending ramus of the mandible.

### Exploration of the genetic variation for modularity and covariation between modules

**Figure 5:**
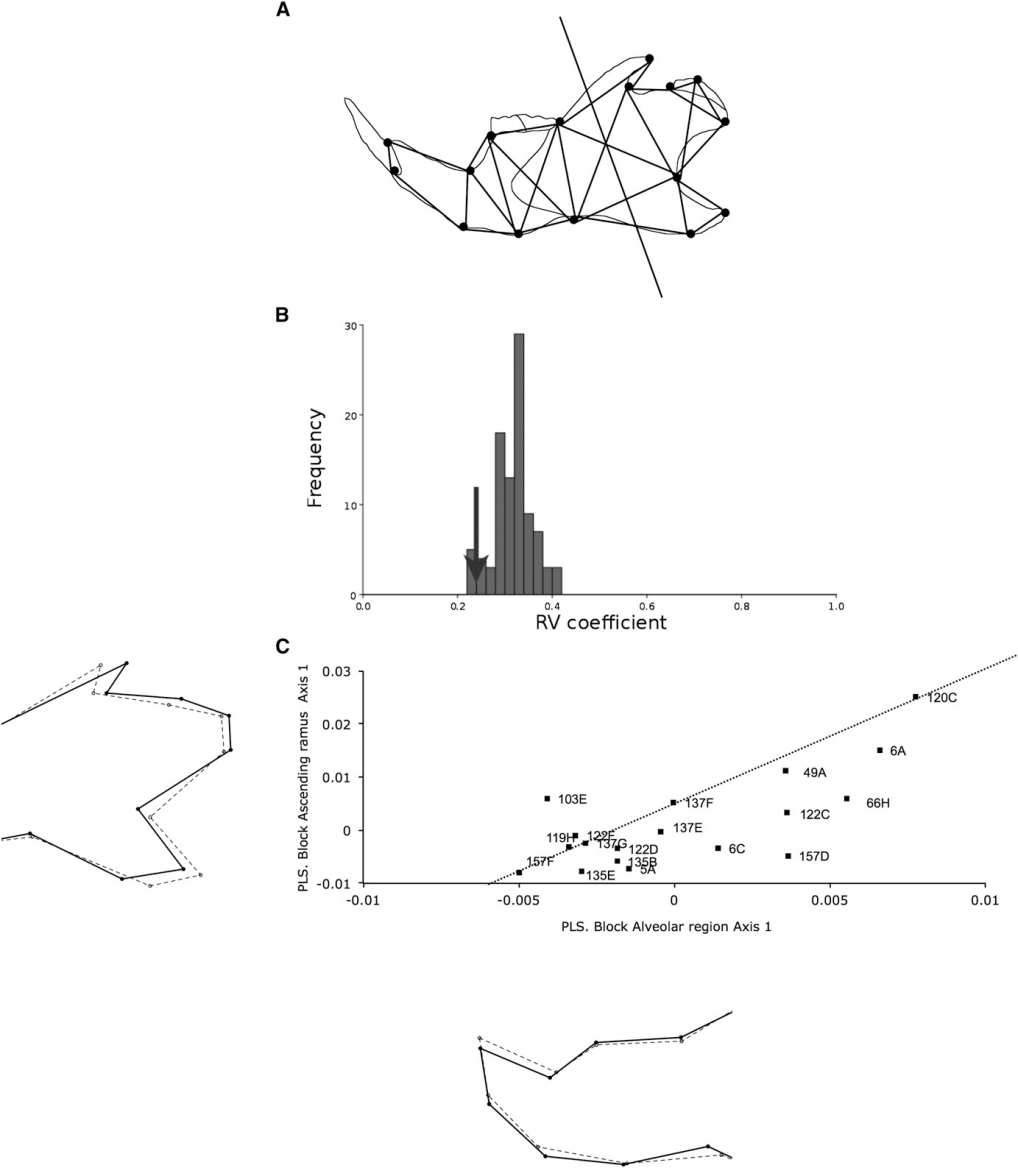
Analysis of modularity in the mandible. (A) Adjacency graph for the mandible. The lines connect the landmarks defined as anatomically adjacent and spatially contiguous. The solid line separates the mandible into two morphogenetic components. (B) Distribution of the RV coefficient for all contiguous partitions. The arrow shows the value of the square trace correlation between the alveolar region and the ascending ramus. (C) Covariation between the two morphogenetic components with the two-block partial least squares (2B-PLS) for all IRCSs. The graph represents the distribution of the IRCSs along the first pair of PLS axis 1, accounting for 54.7% of the total cross covariance. The X-axis represents the alveolar region, and the Y-axis represents the ascending ramus. Correlation between the first pair of PLS axis 1 was drawn by a dashed line. Shape changes drawn outside of the scatter plot were calculated with a multivariate regression between the tangent coordinates after Procrustes superimposition and the first PLS axes. For the shape changes along the first PLS axes, the landmarks are indicated by dots; the solid lines represent the conformation corresponding to the higher value on the PLS axes, whereas the dashed lines represented the shape changes for the lower value on the PLS axis.

To determine whether the genetic variation among the IRCSs is modular, we assessed for modularity using the RV coefficient between two subsets of landmarks: the ascending ramus (landmarks 5–12) and the alveolar region (landmarks 1–4 and 13–16) (Figure 5A). These results are shown in Figure 5B. The RV coefficient for all IRCSs was low to moderate between the ascending ramus and the alveolar region (RV = 0.239) and significantly less than the other partitions (142/6435 full partitions and 4/94 contiguous partitions with respective *P* values of 0.022 and 0.042), suggesting a clear clustering into two modules, the ascending ramus and the alveolar region.

To investigate the degree of covariation between the ascending ramus and the alveolar region, we performed 2B-PLS analysis of these two sets of landmarks on all IRCSs. The results are shown in Figure 5C. The first pairs of PLS axis 1 between the alveolar region and the ascending ramus explained only 54.7% (*P* < 0.0001) of the total covariation, indicating a moderate level of morphological integration between these modules and a different covariance pattern among the IRCSs. Scatter plot analysis using the first pair of PLS axis 1 among the IRCSs indicated that the degree of squared covariance between the two blocks was well distributed upon a line extending from 157F to 120C. This indicates that the IRCSs have a similar pattern of integration, meaning the shape changes in the alveolar region lead to changes in the ascending ramus. Interestingly, we found no specific pattern of covariation with IRCSs carrying the highest SEG genome rate in our dataset (120C, 122D, 6C, 5A, and 49A), suggesting that the increase in SEG segment length or number doesn’t necessarily affect the phenotypic variation in one specific area of the mandible. However, the strains 157D and 66H exhibited more pronounced shape changes in the alveolar region than in the ascending ramus, whereas the QTL localized in the strain 103E increase their effects on the ascending ramus rather than on the alveolar region. Shape changes along the lower score are associated with an elongation of the angular process, an obtuse angle between the coronoid process and the condylar process, and a shortening of the condylar process covarying with an elongation of the incisor alveolus. Higher scores on PLS1 show a shortening of the alveolar process covarying with a short angular process and an elongation of the condylar process and the coronoid process.

## Discussion

### Genetic variation for mandible shape is clustered

The combination of an original resource based on interspecific introgression and morphometric geometrics methods has provided novel insights into the genetic architecture of mandible shape variation and morphological modularity. The goal of this study was to determine whether the genetic variation of mandible shape is modular. Using geometric morphometric techniques on a set of IRCSs, we first quantified the amount of variation using a Procrustres ANOVA. We found that the variation among strains was a major contributing factor to the overall variance (77.5%), suggesting that the variation for shape is predominantly dependent on the introgression of the SEG segments into this set of IRCSs. We then sought to determine the amount of variation for each landmark using the variance component method. We found that the variation for the strain effect, individuals, and fluctuating asymmetry was clustered for over 75% of the total variance to the ascending ramus, suggesting a bipartition of the variation into two morphogenetic components: the alveolar region and the ascending ramus. Comparison from previous genetic analyses (Klingenberg *et al.* 2001, 2004) showed that the QTL effects tended to be stronger for the landmarks in the condylar and the coronoid processes. However, the distinction between the ascending ramus and the alveolar region was not clear (Klingenberg *et al.* 2004). In our study, we have clearly demonstrated a bipartition of the variation into the anterior and posterior modules. It has been reported before that these regions differ functionally (Monteiro *et al.* 2005; Zelditch *et al.* 2008) and developmentally (Atchley and Hall 1991; Depew *et al.* 2005). It has notably been shown that mandibular development is sensitive to the dosage of Dlx genes (Depew *et al.* 2005). For instance, downregulation in the expression of Dlx genes displays a gradient effect, from the distal to the proximal part, on the development of the mandible. It also plays a critical role on the regulation of the skeletal elements of the mandible (Chai and Maxson 2006; Depew *et al.* 2005). At the functional level, studies have suggested that the forces exerted by the masticator muscles on the mandible may have an influence on the growth of the mandible and may affect the variation of these two morphogenetic components (Monteiro *et al.* 2005; Monteiro and Nogueira 2010; Zelditch *et al.* 2008). The species *Mus spretus* and *Mus musculus* have been separated for over 1.5 millions years (Guenet and Bonhomme 2003). *Mus spretus* is endemic to the Mediterranean region (Macholan 1999). Unlike *Mus musculus*, *Mus spretus* inhabits dry areas and feeds mainly on seeds and insects (Palomo *et al.* 2009). Together, the introgression between such divergent species living in a different habitat has introduced a huge phenotypic and genetic polymorphism in the recipient genome. As a result, even with a clustered contribution of SEG genome into C57BL/6 in a number of small-sized chromosomal segments, these IRCSs exhibited a strong phenotypic polymorphism, leading to the discovery of a clustered variation. Interestingly, we have previously demonstrated that the variation of skull shape is clustered using this set of IRCSs (Burgio *et al.* 2009).

### Quantitative trait loci detection for mandible shape

Multivariate analysis of variance indicated that the 18 IRCSs included in this study differed from C57BL/6, suggesting that 42 putative SEG segments could be responsible for this phenotypic variation. Comparison with previously published reports gives a good indication of the conservation of these QTL for shape between species. Using morphometric geometrics, 37 QTL for mandible shape were previously reported (Klingenberg *et al.* 2001, 2004; Leamy *et al.* 2008). Some of the SEG genome regions carried in this set of IRCSs overlapped with these previously described QTL: SH 1.1 (strain 137G); SH 2.2 (strain 120C); SH 6.2 (strains 5A and 49A); SH 6.3 (strains 157F, 137E, 157D, and 122F); SH7.2 (strain 6C); SH 9.1 (strain 103E); SH 12.1 and 12.2 (strain 120C); SH 13.2 (strains 6A and 135B); SH 14.2 (strain 103E); SH 15.1 (strain 49A); and SH 19.1 (strains 103E, 122F, and 6A). Interestingly, 4 IRCSs (strains 66H, 119H, 122C, and 135E) carry 8 different SEG segments in locations not previously described. Out of these 4 lines, the congenic strain 135E carries only one SEG segment on the chromosome 19 (45–60 Mb from the centromere); this novel QTL is therefore called SH 19.2. Moreover, 10 QTL previously described (Leamy *et al.* 2008) were confirmed using this set of IRCSs with a genetic resolution of roughly 10 cM (Burgio *et al.* 2007). Overall, it is difficult to give an exact estimation of the number of the QTL responsible for variation in mandible shape in this dataset due to a partial coverage of the SEG genome (close to 25%). Integration of more strains in this dataset and second-generation crosses between a RCS and B6 will refine these genomic locations and will lead to the discovery of novel QTL for mandible shape.

### Visualization of shape differences between the IRCSs and C57BL/6

The visualization of the shape differences on the mandible using geometric morphometric methods indicated a spatial pattern of variation localized to specific parts of the mandible rather than to the mandible as a whole. The condylar process, the angle, the condylar process, and the incisor alveolus were more likely subject to shape variation. Interestingly, shape differences for the congenic strains (135E, 137E, and 157D) and the IRCSs carrying the highest SEG genome rate (120C, 122D, 6C, 5A, and 49A) were localized on the mandible as a whole. Together, these data suggest a modular pattern of variation as previously reported (Klingenberg *et al.* 2001, 2004). Increasing the SEG genome size doesn’t necessarily affect the shape changes in a specific part of the mandible, suggesting this modular pattern of variation may be unrelated of the SEG genome size.

### Quantitative trait loci effects

To determine whether the QTL effects were additive, we measured the magnitude of the QTL effects using Procrustes distances, and we used correlations across either the SEG segment sizes or the gene density. We found an association between the magnitude of the Procrustes distances and the SEG segment size, indicating an additive effect of these QTL. However, the contribution of dominant *vs.* recessive effects could not be determined, as the percentage of heterozygous SEG alleles in the IRCSs is less than 3%. Nevertheless, this finding is further independent validation of previous studies using chromosome substitution strains (PWD/Ph and C57BL/6) (Boell *et al.* 2011) or crosses of laboratory strains (LG/J and SM/J) (Boell *et al.* 2011)). These findings are consistent and remarkably conserved both among strains and among species as previously found (Boell *et al.* 2011). Interestingly, the comparison of the magnitude of the Procrustes distances with previously published data (Boell *et al.* 2011; Klingenberg *et al.* 2004) demonstrated an average 2-fold increase of the QTL effects compared with the chromosome substitution strain dataset (Boell *et al.* 2011) or a 5- to 6-fold increase compared with a two-generation cross of laboratory strains (Klingenberg *et al.* 2004). This result highlights again the strong effect of the SEG segments on the phenotypic polymorphism. This additivity of loci was not corroborated with the calculation of the correlation across the SEG gene density and the magnitude of the QTL effects. This discrepancy may be explained by a systematic bias that occurred during the recombination events, privileging SEG interval with low recombination events and a high gene density over the inbreeding period of the IRCSs (Noor *et al.* 2001). Alternatively, it may be explained by low statistical power due to a small sample size (18 IRCSs). However, it is difficult at this stage to draw any further conclusion on the additivity of the SEG alleles based on this set of interspecific recombinant congenic strains due to the partial SEG genome coverage with 18 IRCSs.

Current hypothesis argues that genetic architecture of quantitative traits is driven by a small number of loci that with large effects associated with a large number of loci with small effects constituting the majority of the loci (Flint and Mackay 2009). Using this set of interspecific recombinant congenic lines, we showed that the QTL act in an additive manner, suggesting that the genetic variation for shape is an addition of multiple loci on a SEG segment may be close to an infinitesimal model. Alternatively, the genetic architecture for mandible shape may be an assemblage of a multitude of QTL with small effects and a few QTL with large effects. Therefore, only a limited number of IRCSs in this dataset would exhibit a higher than expected Procrustes distance relative to the SEG segment length. Indeed, this was observed for strains 49A and 66H (Figure 3). However, in both models, we only detected a limited number of QTL in relatively large SEG segments (∼10–20 Mb), although the sample size used here was small (362 mice in total), and the coverage of the SEG genome was low (∼25%). Moreover, in the present study, we have not explored the effect of epistasis or dominance on the genetic variation for shape. However, it has been shown before, by using a set of IRCSs on the skull shape (Burgio *et al.* 2009, 2012) or on the mandible and skull shape using different breeding strategies, that epistasis plays a crucial and pervasive effect on controlling the genetic variation for shape (Boell *et al.* 2011; Leamy *et al.* 2002; Wolf *et al.* 2005). Therefore, it is difficult to reach a conclusion on the model of the genetic architecture of mandible shape using this current dataset. Increasing the number of strains and establishing congenic and subcongenic strains will improve the genetic resolution in this set of IRCSs to find out whether the genetic architecture for mandible shape may follow an infinitesimal model. Alternatively, studies with a powerful genetic resolution, such as the Collaborative Cross Consortium (2012), will also improve the statistical power to detect the QTL with small effects and provide novel insights into the genetic architecture of mandible shape.

### Genetic variation for modularity and morphological integration

Using an approach based on the quantification of variation, we have provided some evidence that the variation is clustered in this set of IRCSs. To determine whether the genetic variation for mandible shape is modular, we assessed for modularity using RV coefficient on two *a priori* modules: the alveolar region and the ascending ramus of the mandible. The analysis using the RV coefficient exhibited a significant low level of covariation between the two morphogenetic components on the mandible, suggesting the existence of two modules. This result demonstrated a bipartition model and independently confirms previous results on the mouse mandible (Cheverud *et al.* 1997; Ehrich *et al.* 2003; Klingenberg *et al.* 2004). Interestingly, a previous study on the mouse mandible, which used the squared trace correlation coefficient method, suggested that modular QTL effects for mandible shape were more a question of degrees of covariation rather than dichotomy between the alveolar region and the ascending ramus (Klingenberg *et al.* 2004). In our study, the powerful genetic polymorphism between two highly divergent strains of mice have led to the discovery of a clear modular distinction between these two morphogenetic components.

We also carried out an analysis of the level of covariation using the 2B-PLS technique to determine whether the modules are well integrated within the mandible and to assess the degree of covariation between these two modules. Analysis on all IRCSs revealed that the cross-covariation between the ascending ramus and the alveolar region was moderate (56.7% of the total cross-covariance in the first pair of PLS axes), reaching statistical significance. Visualization on the PLS axis 1 showed a common covariation pattern between the ascending ramus and the alveolar region in most of the IRCSs consisting, for instance, of an elongation of the alveolar region covarying with a shortening of the condylar process, a lengthening of the angular process, and a short coronoid process. Interestingly the covariation for shape for the IRCSs carrying the highest SEG genome rate affected all parts of the mandible.

Overall, this exploration of the morphological modularity and integration suggests that the genetic variation is modular in an integrated scenario. Remarkably, these data also demonstrated that the accumulation of the SEG genome rate in this set of IRCSs increases the magnitude of the QTL effects in an additive, but not in a modular pattern, manner.

In the present work, we have demonstrated the power of interspecific recombinant congenic strains to provide novel insights on the genetic architecture and evolution of mandible shape. This powerful resource has led us to identify novel QTL for mandible shape acting in an additive manner and to demonstrate a genetic clustering of the mandible into two integrated morphogenetic components. Improvement of the statistical power by including more samples and more strains, in combination with the latest genomic technologies leading to the identification of candidate QTL, will provide additional insights into the genetic makeup and the evolution of mandible shape in mouse.

## Supplementary Material

Supporting Information
