# Supplementary material for: Exploration of the Genetic Organization of Morphological Modularity on the Mouse Mandible Using a Set of Interspecific Recombinant Congenic Strains Between C57BL/6 and Mice of the Mus spretus Species
Source: G3 (Bethesda). 2012 Oct 1;2(10):1257–68. doi: 10.1534/g3.112.003285 (PMC3464118; doi:10.1534/g3.112.003285)
Supplement: Supporting Information [file supp_2_10_1257__index.html]

Supporting Information 

# Exploration of the Genetic Organization of Morphological Modularity on the Mouse Mandible Using a Set of Interspecific Recombinant Congenic Strains Between C57BL/6 and Mice of the *Mus spretus* Species

## Supporting Information for Burgio *et al.*, 2012

**Files in this Data Supplement:**

- Supporting Information - Figures S1 and S2 (PDF, 1.8 MB)
- Figure S1 - Genetic map of 137E, 157D and 157F IRCSs indicating the position and sizes of the SEG-derived segments (PDF, 1.0 MB)
- Figure S2 - Genetic map of 6A and 103E IRCSs indicating the position and sizes of the SEG-derived segments (PDF, 824 KB)
