# Supplementary material for: Exploration of the Genetic Organization of Morphological Modularity on the Mouse Mandible Using a Set of Interspecific Recombinant Congenic Strains Between C57BL/6 and Mice of the Mus spretus Species
Source: G3 (Bethesda). 2012 Oct 1;2(10):1257–68. doi: 10.1534/g3.112.003285 (PMC3464118; doi:10.1534/g3.112.003285)
Supplement: Supporting Information [file supp_2.10.1257_FigureS1.pdf]

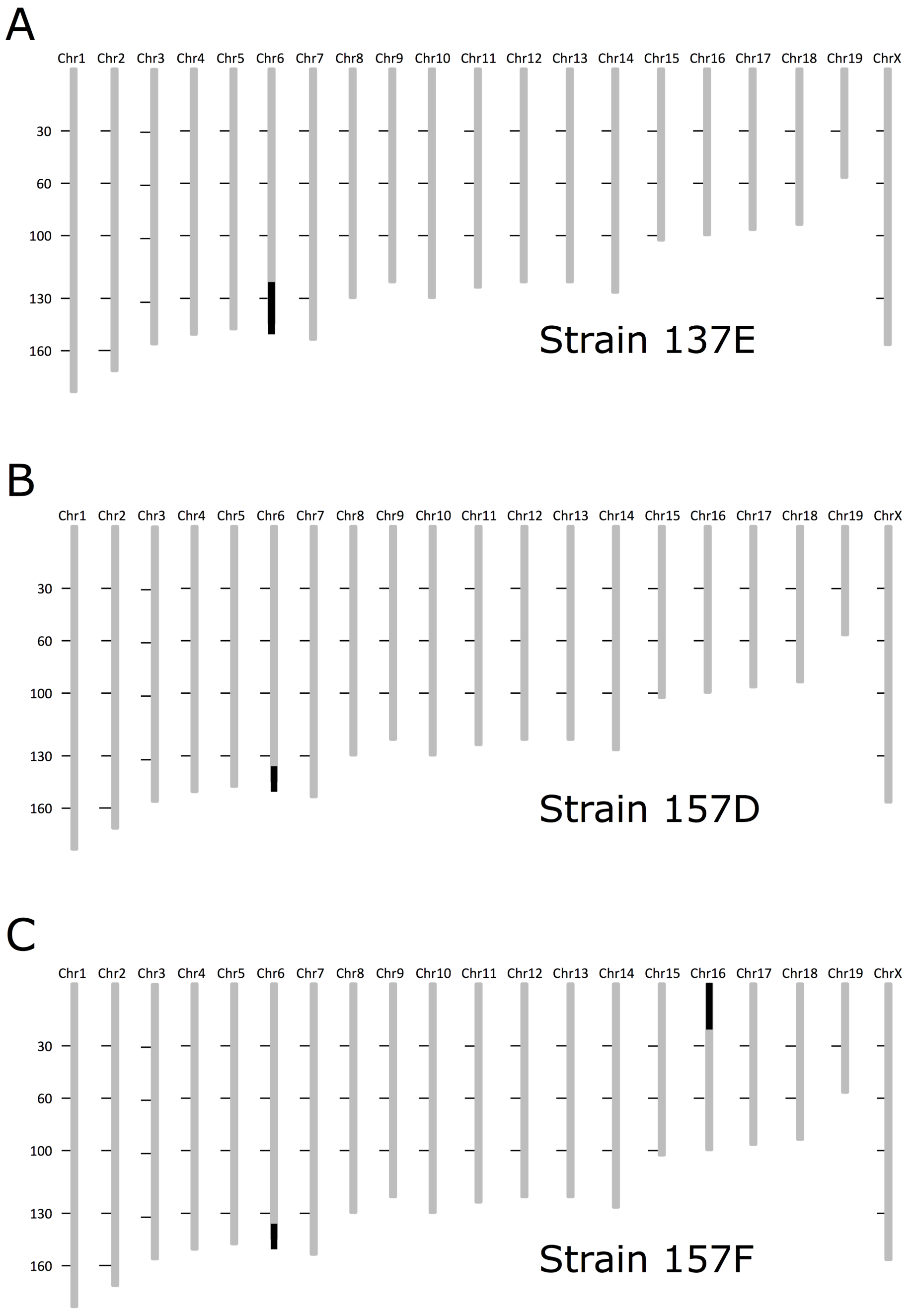

Figure S1 Genetic map of 137E, 157D and 157F IRCSs indicating the position and sizes of the SEG-derived segments. The segments of *Mus spretus* origin are displayed in solid while B6 segments are shaded. The 137E (A) and 157D (B) strains contain one SEG-derived segments on the Chromosome 6 whereas the strain 157F (C) contain 2 SEG-derived segments on the chromosome 6 and 16.
